# Supplementary material for: Experimental evidence for bipolaron condensation as a mechanism for the metal-insulator transition in rare-earth nickelates
Source: Nat Commun. 2018 Jan 8;9:86. doi: 10.1038/s41467-017-02561-6 (PMC5758760; doi:10.1038/s41467-017-02561-6)
Supplement: Supplementary file 1 — Supplementary Information [file 41467_2017_2561_MOESM1_ESM.pdf]

SUPPLEMENTARY NOTE 1: TRADITIONAL DIFFRACTION RESULTS

Rietveld refinement of the neutron diffraction patterns was employed to ensure that the long range structure is consistent with literature for both samples. The diffraction pattern for $LaNiO_3$ is reasonably reproduced using the rhombohedral ($R\overline{3}c$) structure (Supplementary Figure 1a, only data at 300 K is shown) even though it does not reproduce the PDFs described in the text. Whereas lowering the symmetry to the orthorhombic model ($Pnma$) improved the agreement to the PDF, it dramatically worsens the fit to the diffraction data (Supplementary Figure 1b) indicating that the short- and (average) long-range structures are distinct from one another. The orthorhombic model reproduces the data for $NdNiO_3$ well, as expected (Supplementary Figure 1c).

To further test differences in the behavior of the local structure between $LaNiO_3$ and $NdNiO_3$, we have widened the range of small-box refinement to $1.5 - 20$ Å to include not only local polyhedra but also the region where the polyhedra begin to repeat. We first used the high symmetry rhombohedral model. Although this does not fully describe the local structure of either sample at any measured temperature (as shown in the main text), it is still useful as the number of refinable parameters is small, allowing for a straightforward comparison. For example, the x position for oxygen atoms is the only variable atomic position within the unit cell. The x value for $NdNiO_3$ displays a significant discontinuity from 195 K to 170 K while for $LaNiO_3$ it shows linear behavior across the entire temperature regime (Supplementary Figure 2). The abrupt change in the x position again indicates a change in local symmetry in $NdNiO_3$ across the metal-to-insulator (MIT) transition that is absent in $LaNiO_3$.

The ordering of contracted and expanded NiO_6 octahedra described in the main text can also be seen in the second peak in the PDF at ≈ 2.75 Å, which represents O−O correlations (Supplementary Figure 3). As described in the main text for the Ni−O correlation, the O−O peak also initially increases in intensity for both samples as the temperature is lowered but shows a discontinuity for $NdNiO_3$ from 195 K to 170 K caused by the formation of an ordered contraction/expansion that is concomitant with the MIT.

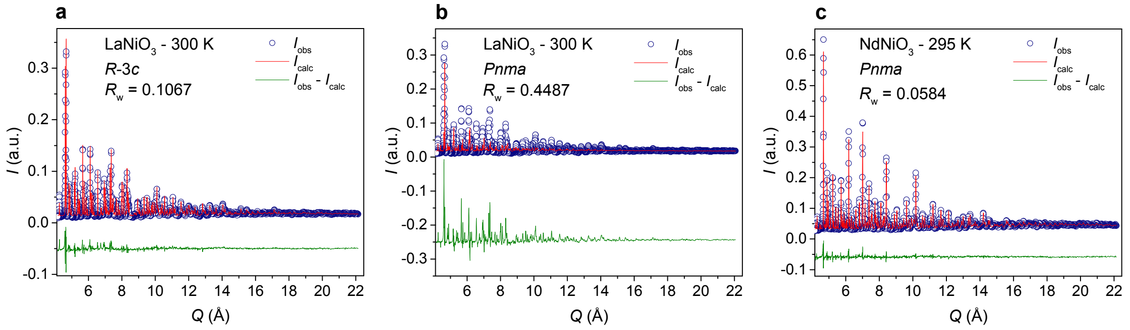

Supplementary Figure 1: Room temperature neutron diffraction results (a) Measured neutron diffraction data for $LaNiO_3$ at 300 K (open circles) refined using the high-symmetry rhombohedral structure (solid red line). The model and data agree reasonably well as indicated by the difference curve between the data and model (green line) and goodness-of-fit parameter (R_w) of 0.1067. (b) Measured neutron diffraction data for $LaNiO_3$ at 300 K (open circles), refined using the lower-symmetry orthorhombic structure (solid red line). The agreement between the model and the data is reduced significantly as indicated by the difference curve (green line) and R_w value of 0.4487. (c) Measured neutron diffraction data for $NdNiO_3$ at 295 K (open circles), refined using the lower-symmetry orthorhombic structure (solid red line). The model and data agree very well as indicated by the difference curve between the data and model (green line) and R_w of 0.0584.

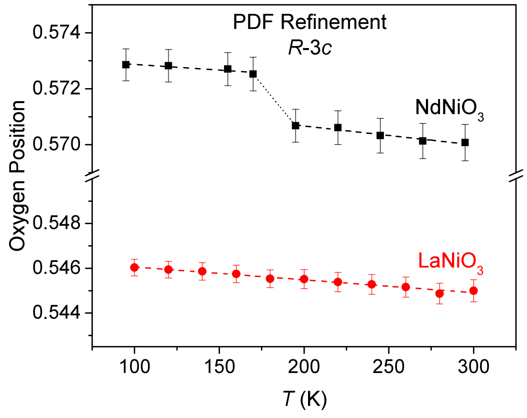

Supplementary Figure 2: Fractional oxygen position (x) determined by small-box PDF refinement for $LaNiO_3$ and $NdNiO_3$ using the high-symmetry, rhombohedral structure.

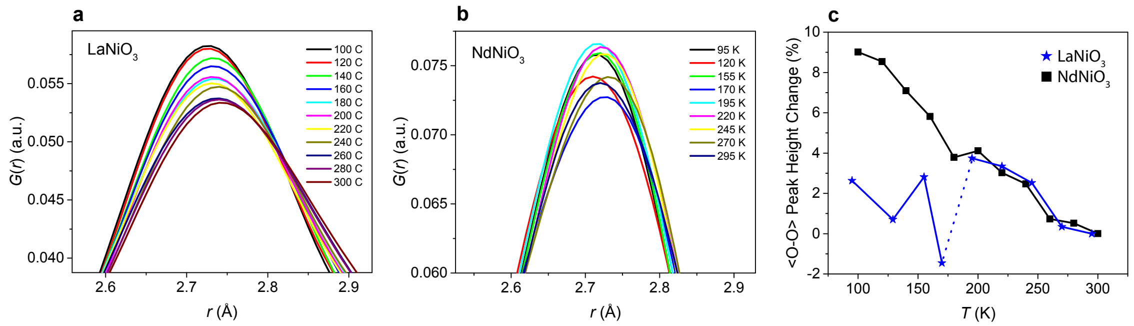

Supplementary Figure 3: Temperature dependence of the PDF peak at ≈ 2.75 Å corresponding to O-O correlations. (a) The O-O PDF peak for $LaNiO_3$ from 300 K to 100 K. A linear baseline was subtracted from the data, which was constant for all temperatures. (b) The O-O PDF peak for $NdNiO_3$ from 295 K to 95 K. A linear baseline has been subtracted from the data which was constant for all temperatures. (c) The peak height of the O-O correlation in the PDF of $LaNiO_3$ (black squares) and $NdNiO_3$ (blue stars) as determined through fitting data in panels (a) and (b) with a Gaussian function. Uncertainties are smaller than the symbols.

SUPPLEMENTARY NOTE 2: ANALYSIS OF THE SLOW CONTRIBUTIONS TO THE CONDUCTIVITY

Given the unusual (slow) nature of charge transport that we observe in the nickelates, it is important to ensure that this mechanism is a bulk characteristic of the sample and not an artifact from an external source (*i.e.* grain boundaries, electrode configuration, ionic conductivity, *etc.*). We can immediately rule out grain boundary hopping, as there is a clear transition in both σ_{dc} and τ in $NdNiO_3$ that coincides with the reported metal-insulator transition and structural transition observed in the present work. Furthermore, the conductivity *decreases* with increasing temperature for metallic $LaNiO_3$, which is expected for metallic conduction but is unphysical for grain-boundary or ionic conduction. Ionic conduction can be further ruled out as a contribution to the signal as we see no evidence of electrode polarization (Supplementary Figure 4). When using oxygen blocking electrodes, ionic conductivity will lead to electrode polarization at low frequencies due to the buildup of mobile oxygen ions at the sample/electrode interface. Supplementary Figure 4 shows such a phenomena for 8 mol% yttria stabilized zirconia, a very good ionic conductor (measured

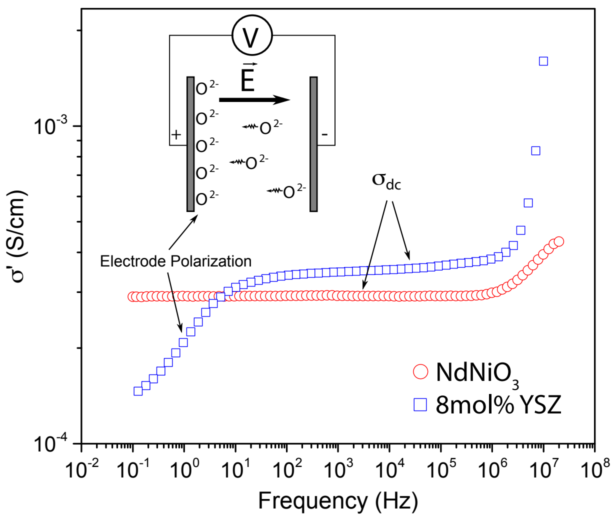

Supplementary Figure 4: Frequency dependence of the real part of complex conductivity (σ') for $NdNiO_3$ (red circles) and yttria stabilized zirconia (YSZ, blue squares) as well as a schematic of electrode polarization (inset). Ionic conduction will lead to a downturn in σ' at low frequencies as mobile oxygen ions "build-up" on ion-blocking electrodes. YSZ, a well-known ionic conductor, shows clear evidence of electrode polarization whereas $NdNiO_3$ shows only dc conduction (σ_{dc}) throughout the entire frequency range.

at 450 °C). Neither sample in the present study show any evidence of electrode polarization at any measured temperature and instead only a flat line at low frequencies corresponding to dc conduction (only $NdNiO_3$ at room temperature is shown).

SUPPLEMENTARY NOTE 3: AIR GAP GEOMETRY

The "air-gap geometry" in the experimental setup serves to reduce the *magnitude* of the total conductivity into the tolerance range of the impedance analyzer (Supplementary Figure 5). Without the air gap, the conductivity is too high for the analyzer to process, and dynamic processes at high frequencies are masked by inductance. Introducing the air-gap essentially forbids the direct "flow" of electrons from one electrode to the other. We stress that this does not affect the measured dynamics of charge transfer or relative conductivity trends. Unlike more traditional 4-probe techniques, BDS measures electrical displacements caused by an alternating electric field. There is no explicit requirement that the electrodes

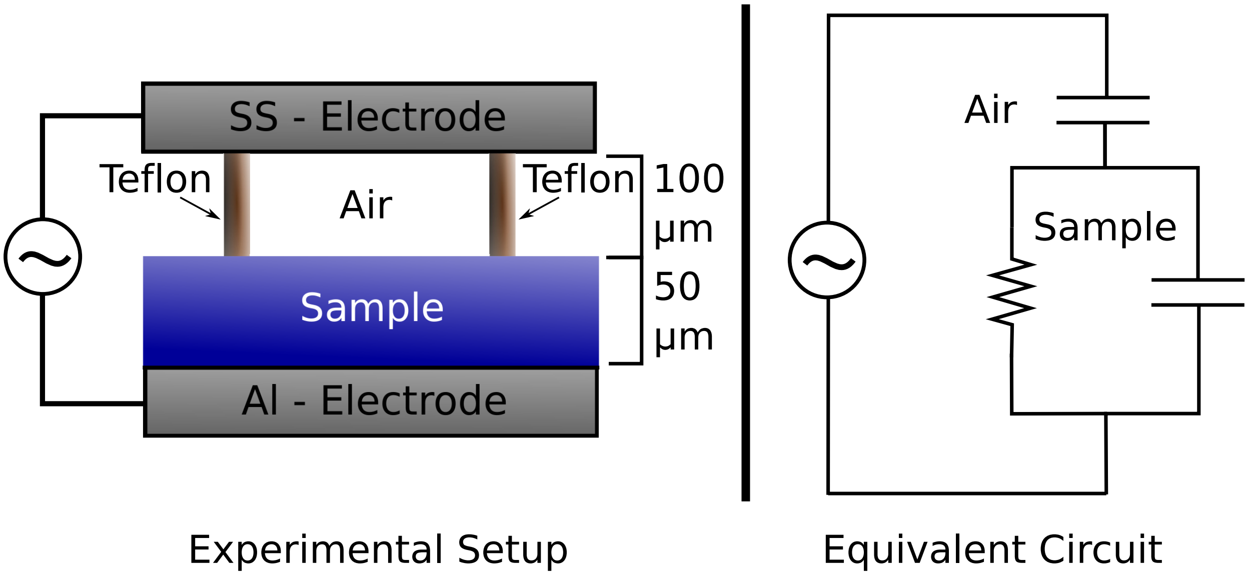

Supplementary Figure 5: Sample cell setup (left) and equivalent circuit (right) for broadband dielectric spectroscopy measurements. The Teflon spacers covered less than 1% of the surface area and were neglected from the equivalent circuit. Because this is a powdered sample, the space between grains will also contribute a capacitance in parallel with the "sample" portion of the circuit which is not shown.

be directly touching the sample (although it is usually desirable to have absolute values that are comparable to 4-probe techniques). The air-gap adds a capacitive layer that is in series with the effective circuit originating from the bulk sample (Supplementary Figure 5). This approach alters the phase of the electric field across the sample but does not change its affinity for displacing differently charged species (*i.e.* electrons *versus* oxygen ions).

SUPPLEMENTARY NOTE 4: ADDITIONAL DATA SET

As mentioned in the main text, we do see some sample variation in the overall time scale and magnitide of the dc conductivity measured within the BDS technique. However, these variations do not change the overall conclusions of the paper as the hopping times are always orders of magnitudes smaller than expected for Drude conduction. In addition, the overall temperature variatious in the extracted quantities are fully reproduceable for variations in the air gap size and between different samples, as shown in Supplementary Figure 6.

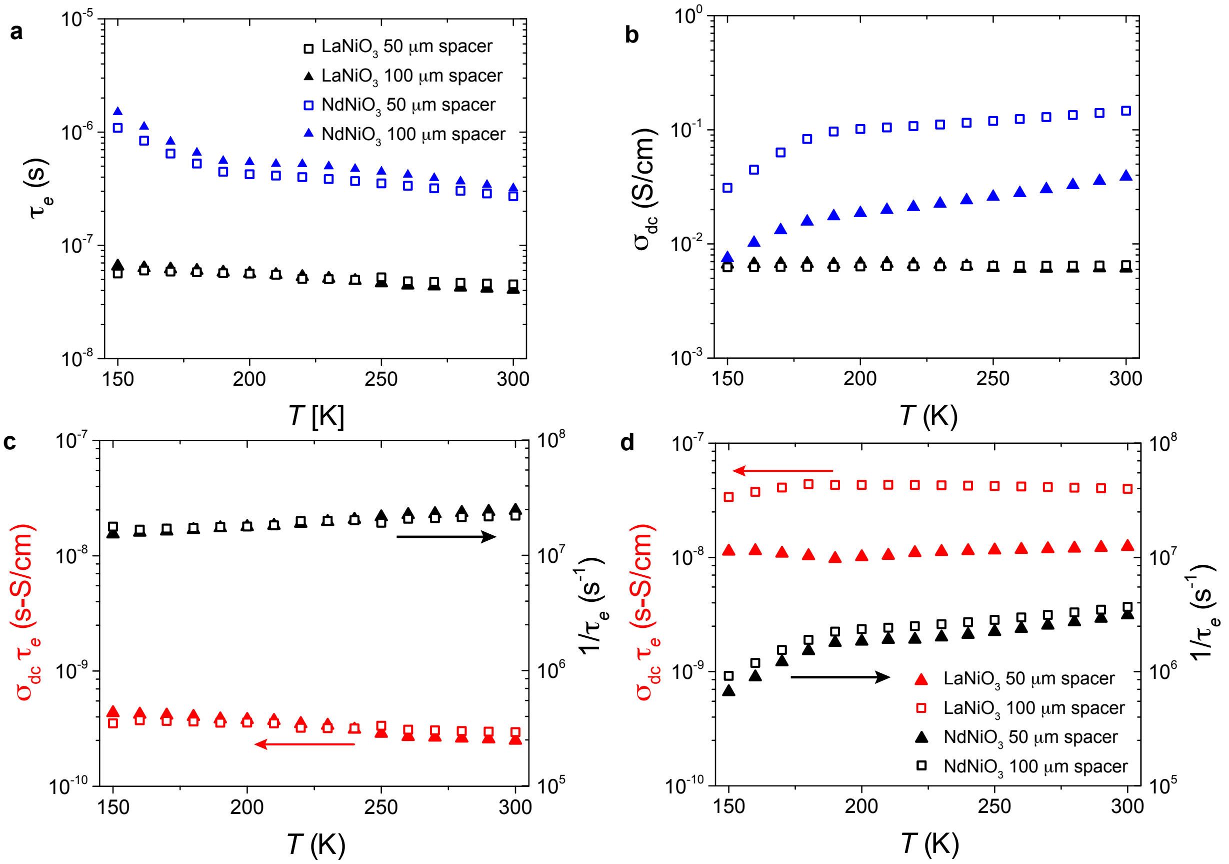

Supplementary Figure 6: Broadband dielectric spectroscopy results obtained from a second set of samples $LaNiO_3$ and $NdNiO_3$ samples. Panels **a**, **b** show the extracted carrier hopping time τ_e and dc conductivity $\sigma_{\rm dc}$ obtained for different air gap geometries. The data shown in this figure was obtained from a completely different set of samples that the data shown in the main text. Panels **c** and **d** show the corresponding temperature dependence of $\tau_e^{-1} \propto \mu_e$ and $\sigma_{\rm dc}\tau_e \propto n$. Note that the powders were repressed between the measurements for different air gap geometries.
